# Prognostic Impact of the SARC-F Score in Gastrointestinal Advanced Cancers

**DOI:** 10.3390/cancers14010010

**Published:** 2021-12-21

**Authors:** Masahiro Matsui, Hiroki Nishikawa, Masahiro Goto, Akira Asai, Kosuke Ushiro, Takeshi Ogura, Toshihisa Takeuchi, Shiro Nakamura, Kazuki Kakimoto, Takako Miyazaki, Shinya Fukunishi, Hideko Ohama, Keisuke Yokohama, Hidetaka Yasuoka, Kazuhide Higuchi

**Affiliations:** 1 The Second Department of Internal Medicine, Osaka Medical and Pharmaceutical University, Takatsuki 569-8686, Japan; masa1987_11_18@yahoo.co.jp (M.M.); masahiro.goto@ompu.ac.jp (M.G.); akira.asai@ompu.ac.jp (A.A.); ushiro.1989@icloud.com (K.U.); oguratakeshi0411@yahoo.co.jp (T.O.); toshihisa.takeuchi@ompu.ac.jp (T.T.); shiro.nakamura@ompu.ac.jp (S.N.); kazuki.kakimoto@ompu.ac.jp (K.K.); takako.miyazaki@ompu.ac.jp (T.M.); shinya.fukunishi@ompu.ac.jp (S.F.); hideko.ohama@ompu.ac.jp (H.O.); hammer_0906@yahoo.co.jp (K.Y.); yh0403.4351@gmail.com (H.Y.); kazuhide.higuchi@ompu.ac.jp (K.H.); 2 The Premier Departmental Research of Medicine, Osaka Medical and Pharmaceutical University, Takatsuki 569-8686, Japan

**Keywords:** gastrointestinal disease, advanced cancer, SARC-F, prognosis, sarcopenia

## Abstract

**Simple Summary:**

There have been few reports with regard to the relevance between the SARC-F score and the prognosis in patients with gastrointestinal advanced cancers, and we aimed to elucidate these issues (*n* = 421, median age = 73 years). During the follow-up period, 145 patients (34.4%) died. The 1-year cumulative overall survival rate in patients with SARC-F ≥ 4 (recommended cutoff point, *n* = 103) and SARC-F < 4 (*n* = 318) was 33.9% and 61.6% (*p* < 0.0001). In the multivariate analysis for the overall survival, total lymphocyte count ≥ 1081/μL (*p* = 0.0014), the SARC-F score ≥ 4 (*p* = 0.0096), Glasgow prognostic score 1 (*p* = 0.0147) and 2 (*p* < 0.0001), ECOG-PS 2 (*p* < 0.0001), and 3 (*p* < 0.0001) and 4 (*p* < 0.0001) were independent predictors. In the receiver operating characteristic curve analysis on the prognostic value of the SARC-F score, the sensitivity/specificity was 0.59/0.70, and the best cutoff point of the SARC-F score was two. The SARC-F score appears to be useful in patients with gastrointestinal advanced malignancies.

**Abstract:**

We sought to elucidate the prognostic impact of the SARC-F score among patients with gastrointestinal advanced malignancies (*n* = 421). A SARC-F score ≥ 4 was judged to have a strong suspicion for sarcopenia. In patients with ECOG-PS 4 (*n* = 43), 3 (*n* = 61), and 0–2 (*n* = 317), 42 (97.7%), 53 (86.9%) and 8 (2.5%) had the SARC-F score ≥ 4. During the follow-up period, 145 patients (34.4%) died. All deaths were cancer-related. The 1-year cumulative overall survival (OS) rate in patients with SARC-F ≥ 4 (*n* = 103) and SARC-F < 4 (*n* = 318) was 33.9% and 61.6% (*p* < 0.0001). In the multivariate analysis for the OS, total lymphocyte count ≥ 1081/μL (*p* = 0.0014), the SARC-F score ≥ 4 (*p* = 0.0096), Glasgow prognostic score (GPS) 1 (*p* = 0.0147, GPS 0 as a standard), GPS 2 (*p* < 0.0001, GPS 0 as a standard), ECOG-PS 2 (*p* < 0.0001, ECOG-PS 0 as a standard), ECOG-PS 3 (*p* < 0.0001, ECOG-PS 0 as a standard), and ECOG-PS 4 (*p* < 0.0001, ECOG-PS 0 as a standard) were independent predictors. In the receiver operating characteristic curve analysis on the prognostic value of the SARC-F score, the sensitivity/specificity was 0.59/0.70, and best cutoff point of the SARC-F score was two. In conclusion, the SARC-F score is useful in patients with gastrointestinal advanced malignancies.

## 1. Introduction

Sarcopenia is characterized by generalized muscle mass loss and muscle functional decline, leading to physical frailty, cachexia, and mortality [1,2]. Malnutrition, reticence, advanced malignancy burden, and persistent inflammatory condition often identified in patients with gastrointestinal disorders are typical clinical characteristics associated with sarcopenia [1,2,3,4,5,6,7,8,9]. Patients with advanced cancer are prone to muscle protein loss due to muscle proteolysis-inducing factors and inflammatory cytokines released by the cancer cells [10]. In addition, psychological anxiety and side effects of treatment for the underlying malignancies can lead to a loss of appetite, and numerous previous studies have reported that sarcopenia is an adverse predictor in patients with advanced cancer [11,12,13]. Cancer cachexia is a metabolic abnormality with a systemic inflammatory response, and it is associated with increased catabolism, such as insulin resistance and increased lipid degradation, as well as increased skeletal muscle degradation [10]. In Japan, anamorelin, a ghrelin (appetite stimulating peptide) receptor agonist, is currently the only drug approved for cancer cachexia [14].

SARC-F (strength (S), assistance walking (A), rising from a chair (R), climbing stairs (C), and falls (F)) is a self-reported questionnaire consisting of five queries with the aim of screening for sarcopenia [15,16]. Subjects are asked to answer to the five questions on a scale of 0 to 2, and the total score is calculated [15]. A SARC-F score ≥ 4 is judged to have a strong suspicion for sarcopenia [15]. The SARC-F score can be linked to physical functional reserve and activity of daily life [17,18]. The SARC-F is recommended for use as an initial screening method for sarcopenia in the current international guidelines [19,20], whereas its lower sensitivity for sarcopenia may be a problem [21,22,23]. Therefore, attempts have been made to combine the SARC-F score with age, body mass index (BMI), and calf-circumference to increase sensitivity for sarcopenia [24,25,26]. The use of SARC-calf for sarcopenia screening is also recommended in the current Asian guidelines [19].

To our knowledge, however, there have been few reports with regard to the relevance between the SARC-F score and prognosis in patients with gastrointestinal advanced cancers [27]. Such research questions may deserve to be clarified, which urged us to perform the present analysis.

## 2. Patients and Methods

### 2.1. Patients and Our Study

In the Second Department of Internal Medicine, Osaka Medical and Pharmaceutical University (OMPU) hospital, sarcopenia risk has been assessed by the SARC-F. As a rule, all hospitalized patients were asked to answer the SARC-F questionnaire upon admission. Between May 2020 and September 2021, 421 advanced malignancy patients with SARC-F scores can be found in our database. Stage III or more severe cancer was defined as advanced cancer. In each patient, the most appropriate therapy was chosen through discussion with gastroenterologists, surgeons, and oncologists based on the current guidelines in each cancer. The primary outcome measure was overall survival (OS). First, the OS rate was compared according to the SARC-F score. Next, univariate and multivariate analyses for the OS were performed. Next, survival analysis was performed according to the disease primary site. Finally, a receiver operating characteristic (ROC) curve analysis for the SARC-F based on the prognosis score was performed.

### 2.2. Glasgow Prognostic Score (GPS)

GPS is calculated by the two serum markers: serum albumin level (reference value = 3.5 g/dL) and C reactive protein (CRP) level (reference value = 1.0 mg/dL), and it is a frequently used prognostic system in patients with advanced malignancies [28,29]. GPS 0 was determined as patients with a serum albumin level > 3.5 g/dL and CRP < 1.0 mg/dL, GPS 2 those with serum albumin level < 3.5 g/dL and CRP >1.0 mg/dL. The remaining subjects were classified as GSP 1.

### 2.3. Statistics

The relationship between two variables was assessed by Pearson correlation coefficient (*r*). For the analysis of the significance of prognostic parameters, continuous parameters were dichotomized at the median value. Kaplan-Meier method was used for the cumulative OS rate and the log-rank test was used for testing. Parameters with a *p* value < 0.05 in the univariate analysis were entered into the multivariate analysis (Cox proportional hazard model). The follow-up period was defined as the period from the date of SARC-F score acquisition to the date of confirmed death or last confirmed survival. ROC analysis based on the prognosis was also done for the area under the ROC (AUC) of the SARC-F score, and the cutoff was adopted as the point where the sum of sensitivity and specificity is maximized. In the data presentation, median value (interquartile range (IQR)) was used. A *p* = 0.05 was set at the significant level by the JMP ver. 15 (SAS Institute Inc., Cary, NC, USA).

## 3. Results

### 3.1. Patient Baseline Features

The baseline features for all study subjects (*n* = 421, 272 men and 149 women, median (IQR) age = 73 (66–79) years) are shown in Table 1. The median (IQR) BMI was 21.0 (18.8–23.6) kg/m^2^. In terms of the disease’s primary site, colorectal cancer was most frequently seen (93 cases, 22.1%), followed by pancreatic cancer (78 cases, 18.5%), esophageal cancer (69 cases, 16.4%), gastric cancer (63 cases, 15.0%), biliary tract cancer (62 cases, 14.7%), and hepatocellular carcinoma (HCC, 55 cases, 13.1%). The median SARC-F score was 1. The SARC-F score 0 was found in 198 cases (47.0%), 1 in 53 (12.6%), 2 in 38 (9.0%), 3 in 29 (6.9%), and ≥4 (highly possible for sarcopenia) in 103 (24.5%). GPS 0 was found in 177 cases (42.0%), GPS 1 in 91 cases (21.6%), and GPS 2 in 153 cases (36.3%). ECOG-PS 0 was found in 201 cases (47.7%), 1 in 78 (18.5%), 2 in 38 (9.0%), 3 in 61 (14.5%), and 4 in 43 (10.2%).

### 3.2. The Cumulative OS Rate According to the SARC-F Score and GPS

**Figure 1:**
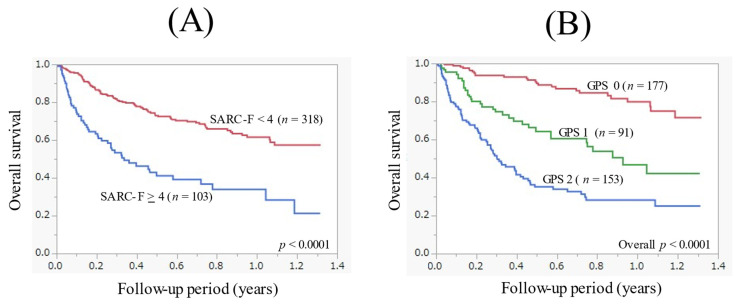
(**A**) Kaplan-Meier curves in patients with the SARC-F score ≥ 4 (*n* = 103) and <4 (*n* = 318). (**B**) Kaplan-Meier curves in patients with the Glasgow prognostic score (GPS) 0 (*n* = 177), GPS 1 (*n* = 91), and GPS 2 (*n* = 153).

The median follow-up period in this study was 0.4 years. During the follow-up period, 145 patients (34.4%) died. All deaths were cancer-related deaths. The 1-year cumulative OS rate for all cases was 55.0%. The 1-year cumulative OS rate in patients with SARC-F ≥ 4 (*n* = 103) and SARC-F < 4 (*n* = 318) was 33.9% and 61.6%, respectively (*p* < 0.0001, Figure 1A). The 1-year cumulative OS rate in patients with GPS 0 (*n* = 177), GPS 1 (*n* = 91), and GPS 2 (*n* = 153) was 79.8%, 47.0%, and 28.2%, respectively (overall *p* < 0.0001, Figure 1B).

### 3.3. The Relationship between the SARC-F Score and ECOG-PS in the Whole Cohort

**Figure 2:**
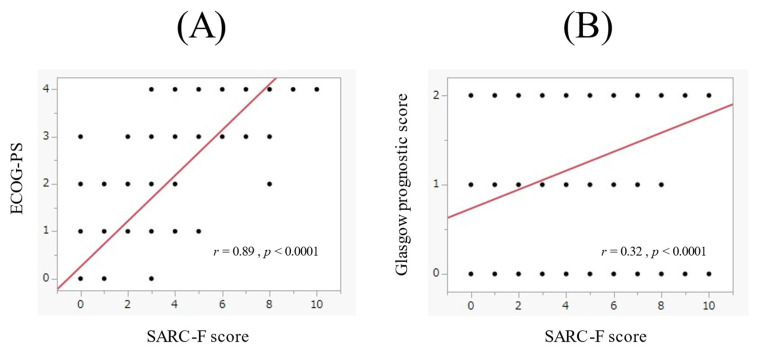
(**A**) The relevance between the SARC-F score and the ECOG-PS. (**B**) The relevance between the SARC-F score and the Glasgow prognostic score.

In the whole cohort, the SARC-F score significantly correlated with EOCG-PS (*r* = 0.89, *p* < 0.0001, Figure 2A). Out of patients with ECOG-PS 4 (*n* = 43), 42 (97.7%) had a SARC-F score ≥ 4. Out of patients with ECOG-PS 3 (*n* = 61), 53 (86.9%) had a SARC-F score ≥ 4. Out of patients with ECOG-PS 0-2 (*n* = 317), 8 (2.5%) had a SARC-F score ≥ 4.

In the whole cohort, the SARC-F score significantly correlated with GPS (*r* = 0.32, *p* < 0.0001, Figure 2B). Out of patients with GPS 2 (*n* = 153), 57 (37.3%) had a SARC-F score ≥ 4. Out of patients with GPS 0 or 1 (*n* = 268), 46 (17.2%) had a SARC-F score ≥ 4.

### 3.4. Uni- and Multivariate Analysis of the OS

In the univariate analysis of the OS, an age ≥ 73 years (*p* = 0.0072), a SARC-F score ≥ 4 (*p* < 0.0001), Hb ≥ 11.9 g/dL (*p* < 0.0001), total lymphocyte count ≥ 1081/μL (*p* < 0.0001), GPS (*p* < 0.0001), and ECOG-PS (*p* < 0.0001) were significant factors (Table 2). In the multivariate Cox regression analysis of the OS, total lymphocyte count ≥ 1081/μL (*p* = 0.0014), a SARC-F score ≥ 4 (*p* = 0.0096), GPS 1 (*p* = 0.0147, GPS 0 as a standard), GPS 2 (*p* < 0.0001, GPS 0 as a standard), ECOG-PS 2 (*p* < 0.0001, ECOG-PS 0 as a standard), ECOG-PS 3 (*p* < 0.0001, ECOG-PS 0 as a standard), and ECOG-PS 4 (*p* < 0.0001, ECOG-PS 0 as a standard) were independent predictors of the OS. Odds ratios (ORs) in each factor are shown in Table 3.

### 3.5. The Cumulative OS Rate Stratified by the SARC-F Score According to the Primary Origin

**Figure 3:**
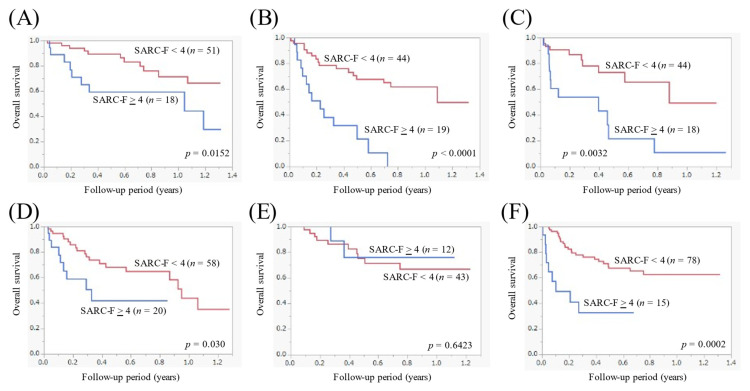
Kaplan-Meier curves in patients with a SARC-F score ≥ 4 and <4 according to the disease primary site. (**A**) Esophageal cancer (*n* = 69). (**B**) Gastric cancer (*n* = 63). (**C**) Biliary tract cancer (*n* = 62). (**D**) Pancreatic cancer (*n* = 78). (**E**) Hepatocellular carcinoma (*n* = 55). (**F**) Colorectal cancer (*n* = 93).

Out of patients with esophageal cancer (*n* = 69), the 1-year cumulative OS rate in patients with the SARC-F ≥ 4 (*n* = 18) and < 4 (*n* = 51) was 59.3% and 71.5%, respectively (*p* = 0.0152, Figure 3A). Out of patients with gastric cancer (*n* = 63), the 1-year cumulative OS rate in patients with the SARC-F ≥ 4 (*n* = 19) and <4 (*n* = 44) was 0% and 62.1%, respectively (*p* < 0.0001, Figure 3B). Out of patients with biliary tract cancer (*n* = 62), the 1-year cumulative OS rate in patients with the SARC-F ≥ 4 (*n* = 18) and <4 (*n* = 44) was 10.8% and 49.3%, respectively (*p* = 0.0032, Figure 3C). Out of patients with pancreatic cancer (*n* = 78), the 1-year cumulative OS rate in patients with the SARC-F ≥ 4 (*n* = 20) and <4 (*n* = 58) was not reached and 43.7%, respectively (*p* = 0.030, Figure 3D). Out of patients with HCC (*n* = 55), the 1-year cumulative OS rate in patients with the SARC-F ≥ 4 (*n* = 12) and <4 (*n* = 43) was 76.2% and 66.9%, respectively (*p* = 0.6423, Figure 3E). Out of patients with colorectal cancer (*n* = 93), the 1-year cumulative OS rate in patients with the SARC-F ≥ 4 (*n* = 15) and <4 (*n* = 78) was not reached and 62.4%, respectively (*p* = 0.0002, Figure 3F).

### 3.6. ROC Analysis on the Prognstic Impact of the SARC-F Score

**Figure 4:**
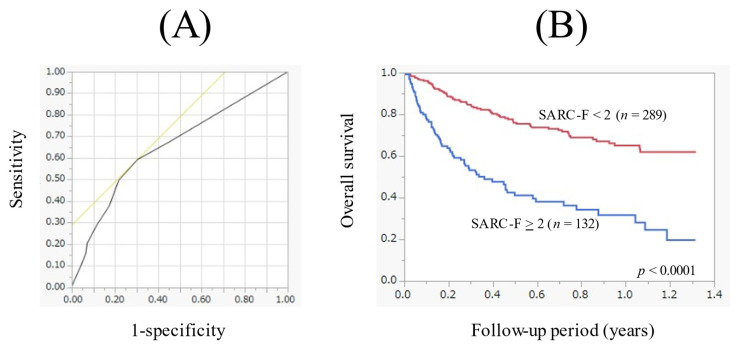
(**A**) Receiver operating characteristics curve based on the prognosis for the SARC-F score. Area under the ROC was 0.66. The sensitivity was 0.59, the specificity was 0.70 and optimal cutoff point of the SARC-F score was 2. (**B**) Kaplan-Meier curves in patients with a SARC-F score ≥ 2 (*n* = 132) and <2 (*n* = 289).

In the ROC analysis on the prognostic impact of the SARC-F score, the AUC was 0.66 (Figure 4A). The sensitivity/specificity was 0.59/0.70, and the optimal cutoff point of the SARC-F score was 2 (Figure 4A). When the cutoff point of the SARC-F score was changed to 2, the 1-year cumulative OS rate in patients with the SARC-F score ≥ 2 (*n* = 132) and <2 (*n* = 289) was 31.6% and 65.0%, respectively (*p* < 0.0001, Figure 4B).

## 4. Discussion

In Japan, due to the spread of the COVID-19 infection, there is a concern that the number of patients receiving cancer screening will decrease and the number of cancer patients who are found in an advanced stage will increase. Although the number of new COVID-19 infections has been decreasing due to the widespread use of COVID-19 vaccines, there is a concern that people, especially the elderly, may be reluctant to receive medical check-ups in a hospital. Thus, an easier screening method without an invasive procedure for the medical check-up may be ideal. As described above, the recommendation in the current international guidelines involves the use of the SARC-F score as an initial screening tool for sarcopenia [12,19,20]. However, to our knowledge, few reports can be found regarding the SARC-F score and the prognosis in patients with advanced gastrointestinal malignancies. If the prognostic impact of SARC-F is clarified, it will be easy to estimate the prognosis of patients with advanced cancer. This is especially significant in facilities with difficulties for assessing muscle mass. We thus conducted the current analysis. We believe that the current results are worth reporting.

In our results, a SARC-F score ≥ 4 was an independent factor for the OS, and in all subgroup analyses except for HCC, patients with a SARC-F score ≥ 4 had significantly worse survival than those with a SARC-F score < 4. These results denoted that the SARC-F is a helpful prognostic scoring system in patients with gastrointestinal advanced malignancies. The reason for the non-significant difference of the OS between the patients with the SARC-F score ≥ 4 and <4 in HCC patients may be attributed to the distribution of the GPS (GPS 0/GPS 1/GPS 2: 5 (41.7%)/5 (41.7%)/2 (16.7%) in the SARC-F score ≥ 4, and 18 (41.9%)/13 (30.2%)/12 (27.9%) in the SARC-F score < 4). A previous meta-analysis demonstrated the prognostic impact of the GPS in HCC patients [30].

In our data, there was a strong correlation between the SARC-F score and ECOG-PS (*r* = 0.89). In patients with PS, 4 (*n* = 43), 42 (97.7%) had a SARC-F score ≥ 4. In patients with PS 3 (*n* = 61), 53 (86.9%) had the SARC-F score ≥ 4. While in patients with PS 0–2 (*n* = 317), only 8 (2.5%) had a SARC-F score ≥ 4. The SARC-F is a questionnaire involving 5 queries as mentioned in the introduction section. The ECOG-PS indicates the degree of limitation in the patient’s daily life. The SARC-F can be an alternative for ECOG-PS. While in our multivariate analysis, total lymphocyte count was an independent predictor as well as EOCG-PS, GPS, and the SARC-F score. The total lymphocyte count reflects immunological function [31]. Exercise can ameliorate sarcopenia through the improvement of immunological function, and thus, exercise can be recommended in patients with advanced cancer [32,33,34]. GPS is a well-established prognostic marker among patients with advanced malignancies [28,29,35]. The ORs for the OS of GPS 1 and GPS 2 in this study were 1.953 (*p* = 0.0147, GPS 0 as a reference) and 3.460 (*p* < 0.0001, GPS 0 as a reference), which were consistent with previous reports [28,29,35]. Although BMI was not significant for the OS in our results, body weight loss was an important factor associated with cancer cachexia [10]. In our cohort, 86 patients (20.4%) had BMI < 18.5 kg/m^2^ (underweight). In such patients, appropriate nutritional intervention may be required to improve prognosis.

In the ROC analysis based on the prognosis for the SARC-F score, AUC was 0.66, the sensitivity/specificity was 0.59/0.70, and the optimal cutoff point of the SARC-F score was 2. When the cutoff point of the SARC-F score was set at 4 (recommended cutoff point), the sensitivity/specificity was 0.38/0.83, indicating a significant decrease in the sensitivity. As described above, a relatively lower sensitivity of the SARC-F score for sarcopenia may be a problem, which is identical to our data [21,22,23]. Hanai et al. reported in their large cohort with chronic liver disease that a SARC-F score = 1 demonstrated a stronger discrimination capability for selecting sarcopenia than a SARC-F score = 4, with an AUC of 0.68, and with the sensitivity/specificity of 0.65/0.68 [36]. In view of these, the cutoff point of the SARC-F score should be carefully reviewed.

We should mention several limitations to the current analysis. First, the study involved a single-center retrospective observational study design. Second, the exact number of patients with a definite diagnosis of sarcopenia was unclear in our data. Third, our study cohort was highly heterogeneous, including a broad spectrum of gastrointestinal advanced malignancies, also creating bias. Fourth, the SARC-F score can vary depending on the patient status, such as malnutrition and dehydration. The patient’s prognosis should therefore be evaluated comprehensively, taking into account factors other than the SARC-F score. Finally, the relatively short follow-up period (median, 0.4 years) in our study should also be mentioned, but the number of deaths was 145 in this study, which can be appropriate for survival analysis.

## 5. Conclusions

The SARC-F score in patients with gastrointestinal advanced malignancies efficiently reflects survival. Appropriate therapeutic interventions should be considered in patients with gastrointestinal advanced malignancies with a higher SARC-F score.

## Figures and Tables

**Table 1:** Baseline characteristics.

Variables	Number or Median (IQR)
Age (years)	73 (66, 79)
Gender, male/female	272/149
BMI (kg/m^2^)	21.0 (18.8, 23.6)
Hb (g/dL)	11.9 (10.4, 13.3)
Serum albumin (g/dL)	3.5 (3.0, 3.9)
CRP (mg/dL)	0.75 (0.19, 4.39)
GPS, 0/1/2	177/91/153
Total lymphocyte count (/μL)	1081 (767, 1490)
ALT (IU/l)	22 (13, 52.5)
Platelet count × 10^4^/mm^3^	21.4 (15.7, 28.0)
eGFR (mL/min/1.73m^2^)	68 (55, 82)
ECOG-PS, 0/1/2/3/4	201/78/38/61/43
SARC-F score, 0/1/2/3/4 or more	198/53/38/29/103
Breakdown of advanced cancers (number (%))	
Esophageal cancer	69 (16.4%)
Gastric cancer	63 (15.0%)
Biliary tract cancer	62 (14.7%)
Pancreatic cancer	78 (18.5%)
Hepatocellular carcinoma	55 (13.1%)
Colorectal cancer	93 (22.1%)
Small intestine cancer	1 (0.2%)

BMI; body mass index, CRP; C reactive protein, GPS; Glasgow prognostic score, ALT; alanine aminotransferase, eGFR; estimated glomerular filtration rate, IQR; interquartile range.

**Table 2:** Univariate analyses of factors linked to the overall survival.

Variables	*n*	*p* Value
Age ≥ 73 years, yes/no	216/205	0.0072
Gender, male/female	272/149	0.0626
BMI ≥ 21 kg/m^2^, yes/no	210/205 *	0.1478
Hb ≥ 11.9 g/dL, yes/no	212/209	<0.0001
Platelet count ≥ 21.4 × 10^4^/mm^3^, yes/no	211/210	0.7554
Total lymphocyte count ≥1081/μL, yes/no	211/210	<0.0001
ALT ≥ 22 IU/l, yes/no	216/205	0.7103
eGFR ≥ 68 mL/min/1.73m^2^, yes/no	213/208	0.0910
SARC-F score ≥ 4, yes/no	103/318	<0.0001
GPS, 0/1/2	177/91/153	<0.0001
ECOG-PS, 0/1/2/3/4	201/78/38/61/43	<0.0001

BMI; body mass index, ALT; alanine aminotransferase, eGFR; estimated glomerular filtration rate, GPS; Glasgow prognostic score, *; missing data, *n* = 6.

**Table 3:** Multivariate analyses of factors associated with the overall survival.

Variables	OR	95% CI	*p* Value
Age ≥ 73 years	1.085	0.763–1.544	0.6491
Hb ≥ 11.9 g/dL	0.830	0.568–1.211	0.3324
Total lymphocyte count ≥ 1081/μL	0.537	0.367–0.787	0.0014
SARC-F score ≥ 4	2.899	1.295–6.489	0.0096
Glasgow prognostic score			
0		Reference	
1	1.953	1.141–3.343	0.0147
2	3.460	2.103–5.691	<0.0001
ECOG-PS			
0		Reference	
1	1.489	0.860–2.580	0.1555
2	4.622	2.691–7.940	<0.0001
3	6.235	2.663–14.601	<0.0001
4	18.82	7.298–48.534	<0.0001

OR; odds ratio, CI; confidence interval.

## Data Availability

Data available on request due to restrictions eg privacy or ethical. The data presented in this study are available on request from the corresponding author. The data are not publicly available due to personal information.

## Abbreviations

BMI	body mass index
OMPU	Osaka medical and pharmaceutical university
OS	overall survival
ROC	Receiver operating characteristic
GPS	Glasgow Prognostic Score
CRP	C reactive protein
AUC	area under the ROC
IQR	interquartile range
HCC	hepatocellular carcinoma
OR	odds ratio

## References

[1] Nishikawa H., Fukunishi S., Asai A., Yokohama K., Nishiguchi S., Higuchi K. (2021). Pathophysiology and mechanisms of primary sarcopenia (Review). Int. J. Mol. Med..

[2] Ganapathy A., Nieves J.W. (2020). Nutrition and Sarcopenia—What Do We Know?. Nutrients.

[3] Chhetri J.K., de Souto Barreto P., Fougère B., Rolland Y., Vellas B., Cesari M. (2018). Chronic inflammation and sarcopenia: A regenerative cell therapy perspective. Exp. Gerontol..

[4] Sieber C.C. (2019). Malnutrition and sarcopenia. Aging Clin. Exp. Res..

[5] Dunne R.F., Loh K.P., Williams G.R., Jatoi A., Mustian K.M., Mohile S.G. (2019). Cachexia and Sarcopenia in Older Adults with Cancer: A Comprehensive Review. Cancers.

[6] Nishikawa H., Shiraki M., Hiramatsu A., Moriya K., Hino K., Nishiguchi S. (2016). Japan Society of Hepatology guidelines for sarcopenia in liver disease (1st edition): Recommendation from the working group for creation of sarcopenia assessment criteria. Hepatol. Res..

[7] Balestrieri P., Ribolsi M., Guarino M.P.L., Emerenziani S., Altomare A., Cicala M. (2020). Nutritional Aspects in Inflammatory Bowel Diseases. Nutrients.

[8] Shaw C. (2021). Management of diet in gastrointestinal cancer. Proc. Nutr. Soc..

[9] Wang H., Yang R., Xu J., Fang K., Abdelrahim M., Chang L. (2021). Sarcopenia as a predictor of postoperative risk of complications, mortality and length of stay following gastrointestinal oncological surgery. Ann. R. Coll. Surg. Engl..

[10] Nishikawa H., Goto M., Fukunishi S., Asai A., Nishiguchi S., Higuchi K. (2021). Cancer Cachexia: Its Mechanism and Clinical Significance. Int. J. Mol. Sci..

[11] Deng H.Y., Chen Z.J., Qiu X.M., Zhu D.X., Tang X.J., Zhou Q. (2021). Sarcopenia and prognosis of advanced cancer patients receiving immune checkpoint inhibitors: A comprehensive systematic review and meta-analysis. Nutrition.

[12] Chen F., Chi J., Liu Y., Fan L., Hu K. (2021). Impact of preoperative sarcopenia on postoperative complications and prognosis of gastric cancer resection: A meta-analysis of cohort studies. Arch. Gerontol. Geriatr..

[13] Trejo-Avila M., Bozada-Gutiérrez K., Valenzuela-Salazar C., Herrera-Esquivel J., Moreno-Portillo M. (2021). Sarcopenia predicts worse postoperative outcomes and decreased survival rates in patients with colorectal cancer: A systematic review and meta-analysis. Int. J. Colorectal. Dis..

[14] Wakabayashi H., Arai H., Inui A. (2021). The regulatory approval of anamorelin for treatment of cachexia in patients with non-small cell lung cancer, gastric cancer, pancreatic cancer, and colorectal cancer in Japan: Facts and numbers. J. Cachexia Sarcopenia Muscle.

[15] Malmstrom T.K., Miller D.K., Simonsick E.M., Ferrucci L., Morley J.E. (2016). SARC-F: A symptom score to predict persons with sarcopenia at risk for poor functional outcomes. J. Cachexia Sarcopenia Muscle.

[16] Ida S., Kaneko R., Murata K. (2018). SARC-F for Screening of Sarcopenia among Older Adults: A Meta-analysis of Screening Test Accuracy. J. Am. Med. Dir. Assoc..

[17] Kim S., Kim M., Won C.W. (2018). Validation of the Korean Version of the SARC-F Questionnaire to Assess Sarcopenia: Korean Frailty and Aging Cohort Study. J. Am. Med. Dir. Assoc..

[18] Tanaka S., Kamiya K., Hamazaki N., Matsuzawa R., Nozaki K., Maekawa E., Noda C., Yamaoka-Tojo M., Matsunaga A., Masuda T. (2017). Utility of SARC-F for Assessing Physical Function in Elderly Patients with Cardiovascular Disease. J. Am. Med. Dir. Assoc..

[19] Chen L.K., Woo J., Assantachai P., Auyeung T.W., Chou M.Y., Iijima K., Jang H.C., Kang L., Kim M., Kim S. (2020). Asian Working Group for Sarcopenia: 2019 Consensus Update on Sarcopenia Diagnosis and Treatment. J. Am. Med. Dir. Assoc..

[20] Cruz-Jentoft A.J., Bahat G., Bauer J., Boirie Y., Bruyère O., Cederholm T., Cooper C., Landi F., Rolland Y., Sayer A.A. (2019). Sarcopenia: Revised European consensus on definition and diagnosis. Age Ageing.

[21] Voelker S.N., Michalopoulos N., Maier A.B., Reijnierse E.M. (2021). Reliability and Concurrent Validity of the SARC-F and Its Modified Versions: A Systematic Review and Meta-Analysis. J. Am. Med. Dir. Assoc..

[22] Lu J.L., Ding L.Y., Xu Q., Zhu S.Q., Xu X.Y., Hua H.X., Chen L., Xu H. (2021). Screening Accuracy of SARC-F for Sarcopenia in the Elderly: A Diagnostic Meta-Analysis. J. Nutr. Health Aging.

[23] Barbosa-Silva T.G., Menezes A.M., Bielemann R.M., Malmstrom T.K., Gonzalez M.C., Grupo de Estudos em Composição Corporal e Nutrição (COCONUT) (2016). Enhancing SARC-F: Improving Sarcopenia Screening in the Clinical Practice. J. Am. Med. Dir. Assoc..

[24] Kurita N., Wakita T., Kamitani T., Wada O., Mizuno K. (2019). SARC-F Validation and SARC-F+EBM Derivation in Musculoskeletal Disease: The SPSS-OK Study. J. Nutr. Health Aging.

[25] Yang M., Hu X., Xie L., Zhang L., Zhou J., Lin J., Wang Y., Li Y., Han Z., Zhang D. (2018). Screening Sarcopenia in Community-Dwelling Older Adults: SARC-F vs SARC-F Combined With Calf Circumference (SARC-CalF). J. Am. Med. Dir. Assoc..

[26] Fu X., Tian Z., Thapa S., Sun H., Wen S., Xiong H., Yu S. (2020). Comparing SARC-F with SARC-CalF for screening sarcopenia in advanced cancer patients. Clin. Nutr..

[27] Behne T.E.G., Dock-Nasimento D.B., Sierra J.C., Rodrigues H.H.N.P., Palauro M.L., Andreo F.O., Silva-The M.B., DE-Aguilar-Nascimento J.E. (2020). Association between preoperative potential sarcopenia and survival of cancer patients undergoing major surgical procedures. Rev. Col. Bras Cir..

[28] Hui D., Paiva C.E., Del Fabbro E.G., Steer C., Naberhuis J., van de Wetering M., Fernández-Ortega P., Morita T., Suh S.Y., Bruera E. (2019). Prognostication in advanced cancer: Update and directions for future research. Support Care Cancer.

[29] Simmons C.P.L., McMillan D.C., McWilliams K., Sande T.A., Fearon K.C., Tuck S., Fallon M.T., Laird B.J. (2017). Prognostic Tools in Patients With Advanced Cancer: A Systematic Review. J. Pain Symptom Manag..

[30] Li M.X., Bi X.Y., Li Z.Y., Huang Z., Han Y., Zhou J.G., Zhao J.J., Zhang Y.F., Zhao H., Cai J.Q. (2015). Prognostic Role of Glasgow Prognostic Score in Patients With Hepatocellular Carcinoma: A Systematic Review and Meta-Analysis. Medicine.

[31] Lei R., Mohan C. (2020). Immunological Biomarkers of COVID-19. Crit. Rev. Immunol..

[32] Suzuki K. (2019). Chronic Inflammation as an Immunological Abnormality and Effectiveness of Exercise. Biomolecules.

[33] Hollingworth T.W., Oke S.M., Patel H., Smith T.R. (2020). Getting to grips with sarcopenia: Recent advances and practical management for the gastroenterologist. Frontline Gastroenterol..

[34] Reginster J.Y., Beaudart C., Al-Daghri N., Avouac B., Bauer J., Bere N., Bruyère O., Cerreta F., Cesari M., Rosa M.M. (2021). Update on the ESCEO recommendation for the conduct of clinical trials for drugs aiming at the treatment of sarcopenia in older adults. Aging Clin. Exp. Res..

[35] Tuomisto A.E., Mäkinen M.J., Väyrynen J.P. (2019). Systemic inflammation in colorectal cancer: Underlying factors, effects, and prognostic significance. World J. Gastroenterol..

[36] Hanai T., Hiraoka A., Shiraki M., Sugimoto R., Taniki N., Hiramatsu A., Nakamoto N., Iwasa M., Chayama K., Shimizu M. (2021). Utility of the SARC-F Questionnaire for Sarcopenia Screening in Patients with Chronic Liver Disease: A Multicenter Cross-Sectional Study in Japan. J. Clin. Med..

